# QuantiFERON®-TB Gold In-Tube Performance for Diagnosing Active Tuberculosis in Children and Adults in a High Burden Setting

**DOI:** 10.1371/journal.pone.0037851

**Published:** 2012-07-12

**Authors:** Michala V. Rose, Godfather Kimaro, Thomas N. Nissen, Inge Kroidl, Michael Hoelscher, Ib C. Bygbjerg, Sayoki G. Mfinanga, Pernille Ravn

**Affiliations:** 1 Department of International Health, Immunology and Microbiology, University of Copenhagen, Copenhagen, Denmark; 2 Clinical Research Centre, University Hospital Hvidovre, Hvidovre, Denmark; 3 Muhimbili Medical Research Centre, National Institute for Medical Research, Dar es Salaam, Tanzania; 4 NIMR-Mbeya Medical Research Programme, Mbeya, Tanzania; 5 Department of Infectious Diseases and Tropical Medicine, Klinikum of the University of Munich, Munich, Germany; 6 Department for Infectious Diseases, University Hospital Odense, Odense, Denmark; Menzies School of Health Research, Australia

## Abstract

**Aim:**

To determine whether QuantiFERON®-TB Gold In-Tube (QFT) can contribute to the diagnosis of active tuberculosis (TB) in children in a high-burden setting and to assess the performance of QFT and tuberculin skin test (TST) in a prospective cohort of TB suspect children compared to adults with confirmed TB in Tanzania.

**Methods:**

Sensitivity and specificity of QFT and TST for diagnosing active TB as well as indeterminate QFT rates and IFN-γ levels were assessed in 211 TB suspect children in a Tanzanian district hospital and contrasted in 90 adults with confirmed pulmonary TB.

**Results:**

Sensitivity of QFT and TST in children with confirmed TB was 19% (5/27) and 6% (2/31) respectively. In adults sensitivity of QFT and TST was 84% (73/87) and 85% (63/74). The QFT indeterminate rate in children and adults was 27% and 3%. Median levels of IFN-γ were lower in children than adults, particularly children <2 years and HIV infected. An indeterminate result was associated with age <2 years but not malnutrition or HIV status. Overall childhood mortality was 19% and associated with an indeterminate QFT result at baseline.

**Conclusion:**

QFT and TST showed poor performance and a surprisingly low sensitivity in children. In contrast the performance in Tanzanian adults was good and comparable to performance in high-income countries. Indeterminate results in children were associated with young age and increased mortality. Neither test can be recommended for diagnosing active TB in children with immature or impaired immunity in a high-burden setting.

## Introduction

The aim of this study was to investigate the potential of the interferon-γ release assay (IGRA) QuantiFERON®-TB Gold In-Tube (QFT) (Cellestis Limited Chadstone, Australia) as a reliable diagnostic tool for physicians in Tanzania, a high TB burden country, with considerable prevalence of HIV and malnutrition, for diagnosing active TB in children.

TB is a major contributor to childhood morbidity and mortality, with children <5 years at highest risk and 40–50% of infected infants developing disease within 1–2 years [1], [2]. However childhood TB is notoriously difficult to confirm, due to its pauci-bacillary nature, and existing tools have limited performance [3].

In TB endemic countries, diagnosis is usually a clinical diagnosis, relying on recognition of clinical features, suggestive chest x-ray (CXR) and if available a positive tuberculin skin test (TST) [4]. However it can be difficult to distinguish between the signs and symptoms of TB, HIV and malnutrition, and CXRs and TST results can be difficult to interpret [5], [6].

QFT relies on *M.tuberculosis* (MTB) specific T-cell responses, measuring levels of interferon-gamma (IFN-γ) released in whole blood in response to stimulation with the MTB specific antigens: ESAT-6, CFP-10 and TB7.7, indicating past or present infection.

IGRAs are generally more specific than TST, since they do not cross-react with the BCG vaccine, *M.avium* or most other non-tuberculous mycobacteria (NTM). IGRAs are increasingly being used worldwide as an alternative to, or in conjunction with, TST [7] for diagnosing latent TB and although not directly diagnostic for active TB, IGRAs are often used by clinicians as an additional tool in the diagnosis of active TB [8]. However there is a lack of evidence of the performance in children, especially in young children in a high burden setting [9].

In this study the performance of QFT was assessed in terms of sensitivity, specificity, indeterminate rates, median IFN-γ levels and risk factor analysis for positive and indeterminate results. Performance of TST for diagnosing active TB was included for comparison. Considering the diagnostic challenges in children and difficulties in assuring a gold standard of confirmed TB, adults with confirmed active TB, from the same location, were included as a measure of contrast.

**Figure 1:**
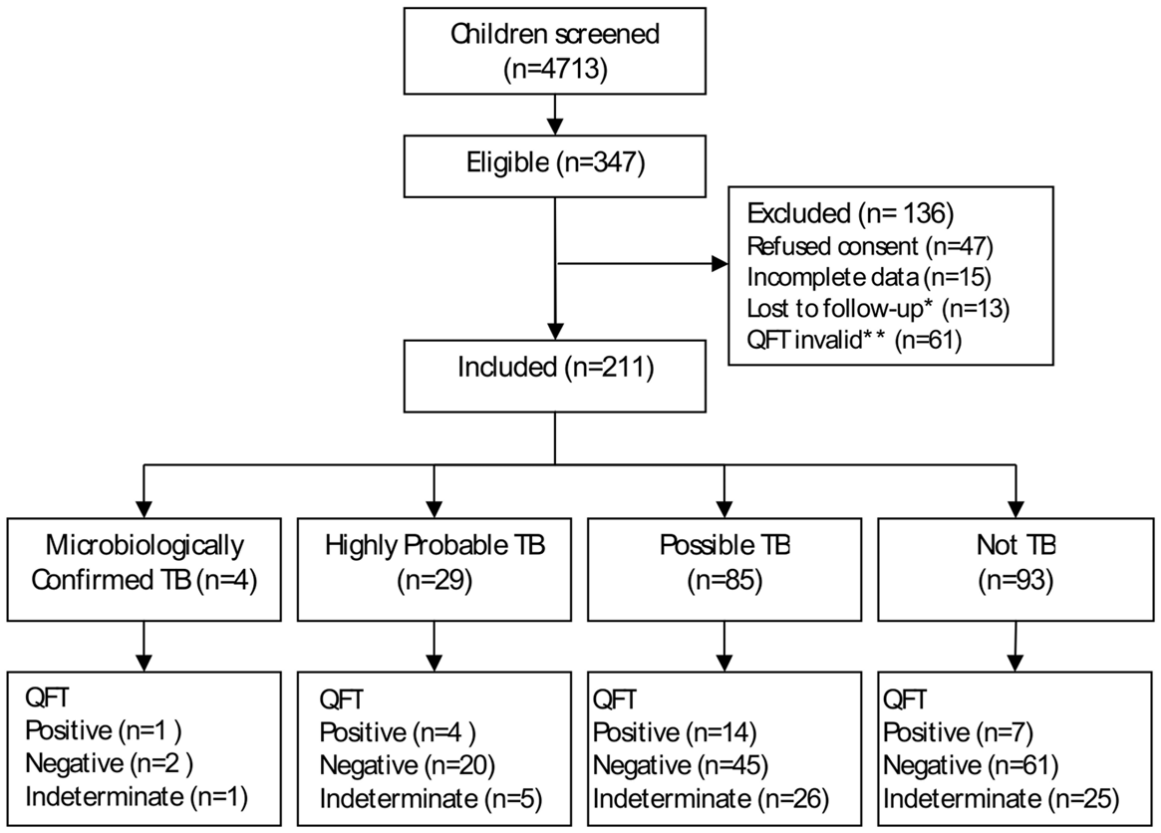
Summary of recruitment and diagnostic classification of children. * Children without follow-up data were excluded since they could not be classified according to the TB classifications. However one child had culture confirmed TB and could therefore be classified without follow-up data. ** The first 61 QFT results were excluded, when the initial QFT analysis showed very poor response in all the QFT tubes, including the mitogen. This was attributed to incorrect storage in a room reaching temperatures above 30°C. Subsequent tubes were all stored at 5–10°C.

**Table 1:** Characteristics of study population.

	Children all		Confirmed		Possible		Not TB		Adults	
	N = 211		N = 33		N = 85		N = 93		N = 90	
**Age** mean yrs (SD)	**4.4**	**(3.8)**	4.7	(3.7)	4.1	(3.9)	4.5	(3.7)	**39.5**	**(14.9)**
**Age groups** n (%)										
<2 yrs	**77**	**(37)**	7	(21)	36	(42)	34	(37)	**–**	**–**
2–4.9 yrs	**53**	**(25)**	13	(40)	21	(25)	19	(20)	**–**	**–**
5–9.9 yrs	**51**	**(24)**	8	(24)	15	(18)	28	(30)	**–**	**–**
>10 yrs	**30**	**(14)**	5	(15)	13	(15)	12	(13)	**–**	**–**
**Male** n (%)	**124**	**(59)**	24	(73)	47	(55)	53	(57)	**72**	**(80)**
**HIV Positive** † n (%)	**78**	**(37)**	18	(55)	38	(45)	22	(24)	**25**	**(28)**
**Z-score** ≤−2 † n (%)	**109**	**(58)**	15	(47)	45	(63)	49	(58)	**–**	**–**
**BMI** <18.5 n (%)	**–**	**–**	–	–	–	–	–	–	**57**	**(63)**
**History contact** † n (%)	**72**	**(35)**	13	(39)	30	(36)	29	(32)	**–**	**–**
**BCG history** n (%)	**196**	**(93)**	30	(91)	81	(95)	85	(91)	**–**	**–**
**BCG scar** n (%)	**192**	**(91)**	28	(85)	75	(88)	89	(96)	**70**	**(78)**
**Clinical TB** * n (%)	**76**	**(34)**	33	(100)	43	(51)	0	0	**90**	**(100)**
**Follow-up status** †n (%)										
Healthy	**152**	**(72)**	31	(97)	28	(33)	93	(100)	**–**	**–**
Still ill	**19**	**(9)**	–	–	19	(22)	0	0	**–**	**–**
Dead	**39**	**(19)**	1	(3)	38	(45)	0	0	**–**	**–**

† HIV test result available for 209 children, weight-for-age Z-score data available for 188 children, history of contact data available for 208 children (reported contact to case of TB in last 2 years), 1 child with confirmed TB did not have follow-up data (follow-up status defined as health status of child 6 months after inclusion into study).

* Clinical TB in children defined as active TB diagnosed by local physician based on clinical examination, CXR and TST. Clinical TB in adults defined as active TB diagnosed by local physician, all included adults had positive Ziehl-Neelsen smear microscopy as well as either positive culture and/or positive fluorescence microscopy.

## Methods

### Ethics Statement

The study protocol was approved by the Tanzanian Medical Research Coordinating Committee (NIMR/HQ/R.8a/Vol IX/584) and was evaluated by the Danish Central Ethical Committee without any objections. Written informed consent was obtained from the immediate caretaker, or next of kin, prior to inclusion, on behalf of children participating in the study. The standards for reporting diagnostic accuracy studies (STARD) criteria were followed in reporting the results.

### Study Setting and Population

The study participants were recruited prospectively at Muheza designated district hospital, Tanga, Tanzania. Muheza is a rural district with a population of 209.480, where 90% are engaged in peasantry, fishery and small-scale business [10]. The TB notification rate in the district in 2009 was 431/100.000 [11].

Children <15 years with TB suspect signs and symptoms were included consecutively from the paediatric ward and outpatients departments, including the HIV, TB, and mother-child clinics. Adults with active TB were included consecutively from the TB clinic. Inclusion criteria are listed in Box S1.

A standardized questionnaire was used to record demographic and clinical details of the participants, including age, weight, height, TB specific signs and symptoms and presence of BCG scar. For the children additional information was collected about history of BCG vaccine, TB exposure, response to prior antibiotic treatment and history of health facility visits.

All the study participants provided blood for QFT and HIV testing. Chest x-rays and TST were performed and either sputum or gastric wash samples were sent for microscopy and culture examination. Follow-up of the children was conducted 2 and 6 months after inclusion, involving a standardised clinical examination and questionnaire concerning health status since last examination. Children who did not return for follow-up were traced within 7–12 months of inclusion.

### Interferon Gamma Release Assay (IGRA)

Venous blood was collected in a syringe and immediately dispensed into the QFT tubes, Nil (negative control coated with saline), TB-Ag (coated with MTB specific antigens) and Mitogen (positive control coated with phytohaemaggluttin), according to the manufacturer’s instructions (Cellestis Ltd). The tubes were taken to the laboratory within 4 hours and incubated at 37°C for 16–24 hours.

Immediately after incubation the samples were centrifuged and the supernatants stored at minus 70°C, until IFN-γ was measured using the QFT ELISA at the NIMR-Mbeya Medical Research Programme laboratory, Tanzania. The results were reported as positive, negative or indeterminate according to the manufacturer’s instructions. In addition the raw quantitative results were recorded.

The results of the first 61 QFT tests revealed that 82% of the results were indeterminate with poor response in all the QFT tubes. Thorough quality assurance of all the test procedures indicated that the tubes had been stored under conditions with temperatures reaching above 30°C. This was considered to be the most likely the reason for the high number of indeterminate results. All the tubes were replaced and new tubes were stored at 5–10°C, until the time they were used.

### Tuberculin Skin Testing (TST)

Two units of purified protein derivate RT23 from Staten Serum Institute, Denmark, were administered intradermally using the mantoux technique recommended by the manufacturer. The transverse diameter of the induration was recorded in millimetres after 48–72 hours. An induration of ≥5 mm was considered positive in HIV positive children and adults whilst an induration of ≥10 mm was considered positive for all others.

### Chest X-Ray (CXR)

The children were chest x-rayed at inclusion. The CXRs were read by three experts; the hospital radiologist and two independent senior radiologists, who were unaware of the clinical status of the child. Using a standardised recording form, the CXRs were classified as either certain TB, highly suggestive TB, uncertain or not TB, based on TB specific or suggestive features. CXRs from 202 children were included.

**Table 2:** Median IFN-γ U/ml in all three Quantiferon TB Gold In-Tube tubes in children and adults.

Children	Negative control	Positive control	TB antigens
	IFN-γ U/ml (IQR)	IFN-γ U/ml (IQR)	IFN-γ U/ml (IQR)
All (TB suspect)	0.15* (0.09–0.35)	2.05* (0.6–7.53)	0.19* (0.1–0.55)
Confirmed TB	0.15* (0.12–0.26)	3.8 (1.08–7.26)	0.17* (0.10–0.48)
Possible TB	0.13* (0.08–0.27)	1.69* (0.44–7.33)	0.17* (0.10–0.56)
Not TB	0.19* (0.09–0.38)	2.26 (0.80–7.61)	0.19* (0.11–0.55)
**Adults**			
Confirmed TB	0.36 (0.22–0.59)	2.63 (1.29–8.09)	3.28(0.99–6.58)

Comparison of median IFN-γ values in the three QFT tubes in children and adults according to diagnostic TB classificaiton. There were significantly lower levels* (p<0.05) in all subgroups of children compared to adults, except in the positive control where median levels in children with confirmed and not TB were not significantly different to levels in adults.

Wilcoxon’s rank-sum test used to test difference between median IFN-γ in children and adults.

IQR: interquartile range.

**Table 3:** Comparison of median IFN-γ responses in all TB suspect children.

		Negative control		Positive control		TB antigens	
		IFN-γ U/ml (IQR)	p-value†	IFN-γ U/ml (IQR)	p-value†	IFN-γ U/ml (IQR)	p-value†
**Age**	<2 yrs	0.14 (0.07–0.21)	–	1.32 (0.4–8.05)	–	0.14 (0.08–0.35)	–
	≥2 yrs	0.16 (0.1–0.44)	0.01	2.37 (1.01–7.33)	0.09	0.21 (0.12–0.64)	0.01
**Sex**	Male	0.17 (0.09–0.45)	–	2.18 (0.73–7.54)	–	0.21 (0.12–0.57)	–
	Female	0.14 (0.09–0.24)	0.08	1.48 (0.49–7.53)	0.27	0.15 (0.09–0.39)	0.04
**HIV status**	Pos	0.14(0.09–0.24)	–	1.4 (0.47–5.61)	–	0.15 (0.09–0.29)	–
	Neg	0.17 (0.09–0.52	0.09	2.49 (0.8–8.89)	0.07	0.2 (0.1–0.84)	0.01
**Z-score**	≤−2	0.14 (0.09–0.37)	–	1.75 (0.6–6.46)	–	0.18 (0.09–0.49)	–
	>–2	0.15 (0.09–0.45)	0.45	2.49 (0.78–9–87)	0.32	0.21 (0.11–0.79)	0.20
**History contact**	yes	0.17 (0.10–0.–36)		2.48 (0.99–7.39)		0.19 (0.10–0.56)	
	no	0.15 (0.09–0.37)	0.56	1.77 (0.53–8.18)	0.37	0.19 (0.10–0.54)	0.77
**Living PTB^+^** *	yes	0.12 (0.1–0.25)		1.91 (0.63–4.35)		0.17 (0.09–0.55)	
	no	0.15 (0.09–0.37)	0.51	1.77 (0.53–8.18)	0.51	0.19 (0.1–0.54)	0.79
**Clinical TB** **	yes	0.15 (0.10–0.41)		2.28 (0.73–7.45)		0.18 (0.1–0.56)	
	no	0.15 (0.08–0.34)	0.69	1.91 (0.52–7.53)	0.56	0.19 (0.1–0.53)	0.68
**Follow–up status**	Healthy***	0.16(0.09–0.39)	–	2.65 (0.95–8.8)	–	0.19(0.11–0.55)	–
	Ill	0.16 (0.08–0.35)	0.59	1.08 ((0.33–2.68)	0.04	0.24 (0.1–0.58)	0.37
	Died	0.11 (0.05–0.2)	0.01	1.02 (0.19–4.39)	<0.01	0.15 (0.07–0.4)	0.18

Comparison of median IFN-γ in subgroups of children, finding lower median levels in response to specific TB antigens in children <2 years, girls and HIV infected and lower median mitogen responses in children who subsequently died.

† Wilcoxon’s rank-sum test used to test differences in median IFN-γ between subgroups of children, p<0.05 considered significant.

IQR: interquartile range.

* Living PTB^+^ defined as living in the same household as a person with smear positive pulmonary TB.

** Clinically diagnosed with TB and put on anti-TB therapy.

*** Subcategory “healthy” used for comparison both for “ill” and “died”.

### Microbiology

Sputum or gastric wash samples were collected from the children on three consecutive mornings. Gastric wash samples were collected in containers containing 100 mg sodium carbonate. Ascites samples were included for 2 patients, whilst no cerebrospinal fluid or lymph node samples were included. The adults were initially diagnosed with smear positive TB at the Muheza district hospital laboratory using the Ziehl-Neelsen staining technique for acid fast bacteria. An extra sputum sample was collected from the adults for confirmation of diagnosis. All the gastric wash and sputum samples were refrigerated, on average 4 days, until they were sent for confirmatory microscopy and culture at the Central TB Reference Lab, Dar es Salaam (CTRL) where auramine staining was used for fluorescence microscopy and Löwenstein-Jensen media for culture. Para-nitrobenzoic acid, which inhibits MTB but not NTMs, was added in positive samples to exclude NTMs.

### TB Diagnosis and Classification

The clinical TB diagnosis in children and the decision to start anti-TB treatment was made by the district TB medical doctor or the medical doctor in charge of the paediatric ward. The study team was not involved other than ensuring that the TB medical doctor was consulted on each case and providing the results of CXR, TST and HIV test. The results of the QFT test were not available to the physicians responsible for diagnosing or treating the children.

Each child was assigned to one of four predefined diagnostic classifications; “Microbiologically confirmed TB”, “Highly probable TB”, “Possible TB” and “Not TB”. The classifications were based on microscopy and culture data, CXR results, clinical examination and follow-up data (Box S2). For data analysis children with microbiologically confirmed TB and highly probable TB were considered to have confirmed TB. Children without follow-up data were excluded, unless they had microbiologically confirmed TB. Adults with either positive culture or positive fluorescence microscopy were classified as confirmed TB.

### Data Management and Statistical Analysis

Data were double-entered into a data entry database using MS-Access (Microsoft Corp, VA, USA) including error, range and consistency check programs. Statistical analyses were performed using Stata™ v 10.0 (Stata Corp, TX, USA). Epi Info™ 3.5.2 (CDC, USA) was used to generate weight-for-age z-scores to assess nutritional status in children.

Indeterminate results were excluded in the sensitivity and specificity analysis as well as in the risk factor analysis for a positive QFT result. Student’s t-test was used to compare means, Wilcoxon’s rank-sum test for comparing medians, using log transformation for data that was not normally distributed.

Logistic regression analysis was used for univariable and multivariable analysis of risk factors’ association with positive and indeterminate QFT results, using 95% confidence intervals to quantify uncertainty. Risk factors that could potentially be associated with the performance of the test were identified from previous studies (age, sex, HIV status, nutritional status and contact history).

Kaplan Meier failure estimates, odds ratios and log-rank test were used to illustrate and quantify the difference in mortality according to QFT results. A p-value of <0.05 was considered statistically significant.

**Table 4:** Sensitivity of Quantiferon TB Gold In-Tube test and Tuberculin Skin test in children and adults.

	QFT positive			TST positive			p-value†
Children	n/all tested*	(%)	95% CI	n/all tested	(%)	95% CI	
Clinical TB diagnosis	11/60	(18.3)	8.4–28.3	8/69	(11.6)	3.9–19.3	0.28
Confirmed TB	5/27	(18.5)	3.4–33.6	2/31	(6)	−2.4–15.3	0.16
Possible TB	14/59	(23.7)	12.7–34.8	7/77	(9.1)	2.6–15.6	0.02
Not TB	7/68	(10.3)	3.0–17.6	3/89	(3.4)	−0.4–7.2	0.03
**Adults**							
Confirmed TB	73/87	(83.9)	76.0–91.8	63/74	(85.1)	76.8–93.4	0.85

Sensitivity of QFT and TST according to TB status in children and adults, showing low sensitivity of both T-cell based test in children, irrespective of TB classification, compared to adults with confirmed TB.

* Sensitivity analysis of QFT excludes indeterminate results.

† Difference between sensitivity in QFT and TST tested using two-sample test of proportion.

## Results

### Study Population

From April 2008 to June 2010 4713 children were screened, 347 children were eligible according to our inclusion criteria and 211 children with TB suspect signs and symptoms were included (figure 1). 107 Ziehl-Neelsen smear microscopy positive adults were screened, 90 with either positive culture and/or positive fluorescence microscopy were included. Characteristics of the study populations are shown in table 1.

Four children were culture positive, two of whom were also fluorescence microscopy positive. In adults 70 were culture and fluorescence microscopy positive, whilst an additional 4 were positive by culture only and 16 by microscopy only.

Based on the above and the criteria in box S2, 33/211 (16%) of children were classified as “confirmed TB”, 85/211 (40%) “possible TB” and 93/211 (44%) “not TB”. All 90 adults were classified as “confirmed TB”.

### TST Results

TST results were available for 197 children (6% positive) and 74 adults (85% positive). Two of four children with TST indurations measuring 5–9 mm were considered TST positive because they were HIV positive. The one adult with an induration of 5–9 mm was HIV negative.

**Table 5:** Risk factors association with positive QFT results in children.

QFT positive (n)						
	(N)	n/N (%)	OR (95% CI)	p-value†	Adj OR^1^ (95% CI)	p-value†
**Age**	≥2 yrs	17/105 (16.2)	1	–	1	–
	<2 yrs	9/49 (18.4)	1.16 (0.48–2.84)	0.73	0.93 (0.34–2.58)	0.89
**Sex**	Female	11/62 (17.7)	1	–	1	–
	Male	15/92 (16.3)	0.90 (0.38–2.12)	0.82	0.95 (0.37–2.43)	0.92
**HIV status**	Neg	19/99 (19.2)	1	–	1	–
	Pos	6/54 (11.1)	0.53 (0.20–1.41)	0.20	0.61 (0.21–1.77)	0.37
**Z-score**	>−2	11/60 (18.3)	1	–	1	–
	≤−2	13/76 (11.9)	0.92 (0.38–2.23)	0.69	1.00 (0.39–2.59)	0.99
**History contact**	No	15/93 (16.1)	1	–	1	–
	Yes	11/60 (18.3)	1.17 (0.50–2.75)	0.72	1.37 (0.55–3.41)	0.49
**Living PTB+** *	No	15/93 (16.3)	1	–	1	–
	Yes	7/27 (25.9)	1.82 (0.65–5.06)	0.25	2.83 (0.89–9.03)	0.08

Risk factor analysis using logistic regression analysis found no association between known risk factors and a positive QFT result in children.

OR: unadjusted odds ratio from univariable analysis.

Adj OR: adjusted odds ratio, adjusted for age, sex, HIV, z-score and contact TB case.

† p-value for the odds ratios.

* Compares living with a case of smear positive TB case to those reporting no contact at all.

**Table 6:** Risk factors association with indeterminate QFT results in children.

QFT indeterminate (n)						
	(N)	n/N (%)	OR (95% CI)	p-value†	Adj OR (95% CI)	p-value†
**Age**	≥2 yrs	29/134 (21.6)	1	–	1	–
	<2 yrs	28/77 (36.4)	2.07 (1.11–3.85)	0.02	2.39 (1.22–4.68)	0.01
**Sex**	Female	25/87 (28.7)	1	–	1	–
	Male	32/124 (25.8)	0.86 (0.47–1.59)	0.64	0.79 (0.40–1.54)	0.49
**HIV status**	Neg	32/131 (24.4)	1	–	1	–
	Pos	24/78 (30.8)	1.38 (0.74–2.57)	0.32	1.28 (0.63–2.63)	0.50
**Z-score**	>−2	19/79 (24.1)	1	–	1	–
	≤−2	33/109 (30.3)	1.37 (0.71–2.65)	0.35	1.09 (0.53–2.21)	0.82

Risk factor analysis using logistic regression analysis found increased odds of an indeterminate result in children <2 years.

OR: unadjusted odds ratio from univariable analysis.

Adj OR: adjusted odds ratio, adjusted for age, sex, HIV and z-score.

† p-value for the odds ratios.

### QFT Results

QFT results were positive in 26 (12%), negative in 128 (61%) and indeterminate in 57 (27%) children. Amongst adults 73 (81%) were QFT positive, 14 (16%) negative and 3 (3%) indeterminate. One of the indeterminate results in children was due to high negative control (>8 U/ml), the remaining indeterminate results in children and adults were due to low response in the positive control.

The median levels of IFN-γ were significantly lower in children compared to adults, especially in response to the TB antigens (table 2). Even children with a positive QFT result had a lower median IFN-γ after antigen stimulation (2.08 U/ml) than adults with a positive QFT (4.09 U/ml), p = 0.03 (data not shown). The median IFN-γ in response to TB antigens was significantly lower in children under 2 years, in girls and HIV positive children (table 3). Amongst children with a positive QFT test, HIV positive children still had a significantly lower median IFN-γ response to TB antigens than HIV negative children (data not shown). Children who died before discharge or during the follow-up period, as well as those who were still ill at 6 month follow-up had lower median IFN-γ in the positive control at base-line compared to those who had regained their health.

**Table 7:** Risk factors association with positive QFT result in adults.

QFT positive (n)						
	(N)	n/N (%)	OR (95% CI)	p-value†	Adj. OR (95% CI)	p-value†
**Age** years*	–		1.0 (0.96–1.04)	0.93	0.10 (0.95–1.04)	0.89
**Sex**	Female	11/16 (68.8)	1		1	
	Male	62/71 (87.3)	3.13 (0.88–11.12)	0.08	2.07 (0.49–8.81)	0.33
**HIV status**	Neg	57/63 (90.5)	1		1	
	Pos	16/24 (66.7)	0.21 (0.06–0.70)	0.01	0.25 (0.07–0.92)	0.04
**BMI**	≥18.5	28/33 (84.9)	1		1	
	<18.5	45/54 (83.3)	0.89 (0.27–2.94)	0.85	0.81 (0.23–2.84)	0.74

Risk factor analysis using logistic regression analysis found lower odds of a positive QFT result in HIV infected adults.

OR: odds ratio from univariable analysis.

Adj. OR: adjusted odds ratio adjusted for age, sex, HIV infection and BMI.

* Odds ratio calculated as per year increase in age.

† p-value for the odds ratios.

**Table 8:** Risk factors association with indeterminate QFT result in adults.

QFT indeterminate (n)						
(N)		n/N (%)	OR (95% CI)	p-value†	Adj. OR (95% CI)	p-value†
**Age** years*		–	1.06 (0.99–1.15)	0.10	1.10 (0.99–1.22)	0.07
**Sex**	Female	2/18 (11.1)	1	–	1	–
	Male	1/72 (1.4)	0.11 (0.01–1.32)	0.08	0.02 (0.00–1.28)	0.07
**HIV status**	Neg	2/65 (3.1)	1	–	1	–
	Pos	1/25 ((4)	1.31 (0.11–15.15)	0.83	0.47 (0.01–17.02)	0.68
**BMI** **	≥18.5	0/33	–	–	–	–
	<18.5	3/57 (5.3)	–	–	–	–

Risk factor analysis using logistic regression analysis found no association between suspected risk factors and an indeterminate QFT result in adults.

OR: odds ratio from univariable analysis.

Adj. OR: adjusted odds ratio adjusted for age, sex, HIV infection and BMI.

* Odds ratio calculated as per year increase in age.

** Logistic regression analysis not possible for association between BMI and indeterminate result, since there are no indeterminate results in those with BMI ≥18.5.

† p-value for the odds ratios.

### Sensitivity of QFT and TST for Diagnosing Active TB

Amongst children, clinically diagnosed with TB and started on anti-TB treatment by the local physician, we found a sensitivity of QFT and TST of only 18% and 12% respectively. Amongst children with “confirmed TB” we found a sensitivity of QFT and TST of 19% and 6% respectively. The sensitivity of QFT and TST in children with “possible TB” was 24% and 9%, and in children with “not TB” 10% and 3% (table 4).

The specificity of QFT and TST in children was 90% and 98% respectively. The positive predictive value of QFT and TST in children for having confirmed TB was 42% and 40%, whilst the negative predictive value was 73% and 75% respectively.

In contrast, the diagnostic sensitivity of QFT and TST amongst adults with confirmed TB was notably higher than in children at 86% and 89% (table 4). Sensitivity of QFT was significantly lower in HIV positive adults 67% (16/24), compared to HIV negative adults 91% (57/63), p = 0.007 (data not shown). The sensitivity of TST in adults was not affected by HIV status. The specificity in adults was not calculated since the inclusion criteria was confirmed TB.

**Figure 2:**
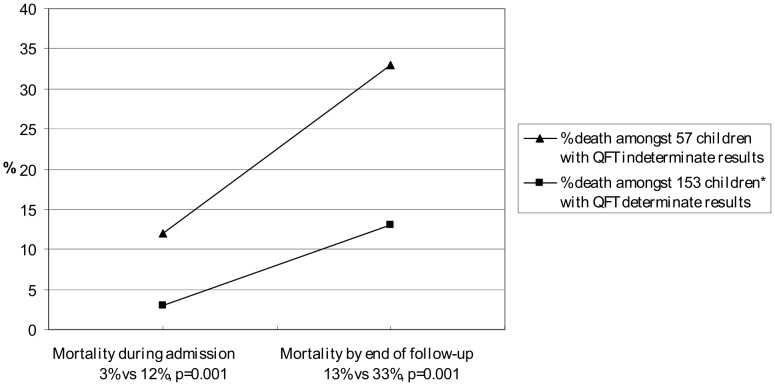
Childhood mortality according to Quantiferon TB Gold In-Tube result. Childhood mortality recorded during admission and at 6 month follow-up, according to QFT results. Children who did not attend scheduled follow-up at 6 months were traced and follow-up visits were conducted in their homes within 7–12 months of inclusion. Both the mortality during admission and the overall mortality in children with an indeterminate QFT result was significantly higher than in children with a determinate QFT result, p<0.001. * One of 154 children with a determinate QFT result did not have follow-up data.

### Risk Factors Association with Positive and Indeterminate QFT Results

Logistic regression analysis was used in order to identify possible causes for the low positivity rate and high indeterminate rate of QFT in children (Table 5 and 6).

Neither sex, malnutrition nor HIV infection, were associated with either positive or indeterminate QFT results in the unadjusted or adjusted analysis. Surprisingly, history of TB contact was not associated with a positive QFT result, even after adjusting for confounders in the multivariable analysis.

Age seemed to influence the test performance as children <2 years were more likely to have an indeterminate result (adjusted OR 2.39, p = 0.01).

In adults HIV infection was negatively associated with a positive QFT result (adjusted OR 0.25, p = 0.04) (table 7), whilst we found no effect of age, sex or HIV status when analysing risk factors for an indeterminate result (table 8). We were unable to determine association with BMI as none with a BMI>18.5 had an indeterminate result.

### Mortality

Eleven children died during admission, whilst an additional 28 children died during the follow up period, resulting in an overall mortality of 19%. An indeterminate QFT result at baseline was associated with subsequent high childhood mortality (adjusted OR 3.87, p = 0.003, data not shown) and the cumulative mortality was significantly higher in children with an indeterminate QFT result (33%) compared to a determinate result (13%), p<0.001 (figure 2).

## Discussion

The main findings of this study were a low sensitivity of both QFT and TST for diagnosing active TB in children as well as a high QFT indeterminate rate which was associated with high subsequent mortality.

We found a surprisingly low sensitivity of QFT in children with confirmed TB (19%) compared to adults with confirmed TB (84%). The sensitivity of TST was similarly poor in children (6%) compared to adults (85%). Using other criteria for diagnosing TB, such as the clinical TB diagnosis or by including children with possible TB in the group of TB cases, did not alter the overall picture of an extremely low sensitivity. In addition the positive and negative predictive values of QFT in children were low.

We found a high specificity (≥ 90%) for both QFT and TST, either suggesting that false positive results due to latent TB or BCG vaccination did not influence the performance of the tests or reflecting the overall poor performance of the test, with low proportions of positive results in this population.

The few existing previous paediatric studies have reported QFT sensitivity for active TB varying from 50%–100% [12]. To our knowledge only one other study has evaluated QFT in a high burden setting and they found only 8 children with confirmed TB and a sensitivity of 63% [13]. Studies evaluating T-Spot®-TB, another commercially available IGRA (Oxford Immunotec, Abingdon, UK), in children in a high endemic setting are equally few, one study in South Africa study with 10 culture confirmed cases of TB, found a sensitivity of 50% [14].

We found only 12 children with a positive TST, which is surprising given that 72 (35%) reported contact to person with TB and 92.9% were BCG vaccinated. The sensitivity of TST (6%) was even lower than QFT.

Infants are known to have poor production of cytokines, such as IFN-γ, even beyond infancy [15] and there is evidence that an effective antigen specific response to the MTB infection is delayed and less effective in infants compared to adults [16] which could explain our comparatively low positivity rate. Not only young age [13] but also HIV infection [17] and malnutrition, [18] were expected to affect test sensitivity, due to immature immune response or suppression of T-cell response. We found no clear association in our risk factor analysis between these factors and the QFT positivity rate, possibly due to small sample size, however we did find reduced IFN-γ responses in children <2 years, HIV infected and children who were still ill or had died by 6 month follow-up. Children in low-income settings often present with severe TB after a long period of illness, and consequent suboptimal immune response [19], [20]. These findings suggest that the performance of QFT as well as TST, in these children with chronic and severe illness, were affected by immature or impaired immunity and progressive exhaustion of T-cells ability to react adequately to antigen response [21].

Due to the lower levels of IFN-γ in response to specific antigens seen in HIV infected and young children it has been suggested that a lower cut-off value for a positive QFT for children may be relevant [22]. However applying a cut-off of 0.26 U/ml to our data only increased the number of positives by two, both of whom were classified “not TB”. Thus our study does not indicate added value of a lower cut-off in terms of sensitivity of QFT for active TB.

The poor sensitivity of, not only QFT but also TST, could also be explained by misclassification and over-diagnosis of TB however we included microbiological confirmation, good clinical response to treatment, in addition to either typical CXR or clinical features, which is in accordance with the recommendations of the recent expert consensus report on evaluating TB diagnostics in children [23].

Like other studies including TB suspect children we found only a small proportion of culture confirmed cases (2%). In the four culture confirmed cases of TB there was only one QFT and one TST positive result (in the same child), indicating that, rather than a classification problem, the performance of T-cell based assays in this particular group of children is suboptimal. Two of the remaining children with culture confirmed TB, were both HIV positive and severely malnourished, and it reasonable to conclude that they were not able to mount an adequate immune response.

Our results highlight the difficulties in diagnosing TB in children and we cannot conclude whether QFT suffers from extremely low sensitivity in young children with active TB in a high burden country or whether TB was over-diagnosed in these very sick children. However in light of the review by Mandalakas et al [12] which found that sensitivity of IGRAs and TST was lower in children <5 years, HIV positive children and in low-middle income countries, our finding of low sensitivity is not unlikely.

In adults we found a sensitivity of 84% which compares to the pooled sensitivity of 80% in a recent review of IGRA studies [8] and in line with another Tanzanian study [17] and the review by Metcalfe [24], we also found a lower sensitivity amongst HIV positive adults.

We found a high indeterminate rate in children (27%) compared to adults (3%) (p<0.01) with higher rates amongst children <2 years and in children who subsequently died after inclusion. Surprisingly our data did not support a significant association with HIV infection or malnutrition possibly due to low numbers.

Indeterminate rates in other paediatric QFT studies range from 0–35% [13], [25], high rates correlated with young age [26], immune suppression [25] and malnutrition [27]. Thomas et al [27] found, in a study in Bangladesh that indeterminate results were associated not only with malnutrition but also with helminth infection, suggesting that children in a generally poor nutritional state with added infections are less likely to have a determinate QFT result. Hook worm and schistosomiasis are known to be highly prevalent amongst school children in the study area [28] and we can reasonably assume that many of the study participants were co-infected, which may have influenced the performance of QFT [29].

In our study 58% of children were defined as being malnourished. Malnutrition leads to an impaired cellular immunity which could affect the performance of the IGRA tests [18], [30] however several studies conducted in populations with significant malnutrition [13], [31], including the current study, did not find malnutrition to be associated with reduced positivity rates or increased indeterminate results. It is possible that weight-for-age z-score is not the optimal measure for assessing the effect of malnutrition on IGRA responses. A study of Peruvian adults found that malnutrition as measured by corrected arm muscle area, but not BMI or body fat, was associated with decreased TST positivity [32].

No previous paediatric studies have shown that an indeterminate result is a risk factor for death. To our knowledge only one study in TB-suspect HIV positive adults in Uganda has shown that mortality was higher in those with an indeterminate T-SPOT.TB result [33]. It is reasonable to hypothesise that the children who died after inclusion were severely ill and therefore unable to mount an appropriate immune response. Thus, we suggest that the care for children with indeterminate results should be intensified with extensive diagnosis and treatment.

We found a low indeterminate rate of 3% amongst adults. Even amongst the HIV positive adults, the indeterminate rate was only 4% which contrasts to other studies, including HIV infected participants, where indeterminate rates reach as high as 21% [34]. HIV infection is known to be associated with lower positivity rates [35] and our data support these findings.

The samples of both children and adults were taken and processed concurrently, at the same hospital using the same staff, procedures and equipment. As such, it is most unlikely that the poor performance in children was due to technical errors.

Our study suggests that T-cell based assays, such as QFT and TST, have poor performance in children with immature or impaired immune systems and that indeterminate results are a predictor for death. With a sensitivity of 19% amongst children with confirmed TB and indeterminate rate of 27%, QFT is not the answer for providing clinicians with a reliable tool for diagnosing childhood TB in a high burden setting. This conclusion is in line with recent reports that underline that IGRA cannot be used as a test to exclude active TB in children [36], that use of IGRAs is not recommended in children under 5 years [7] and according to WHO should not be used in low and middle income countries at all [37]. Whether low sensitivity is due to poor antigen response, misclassification or over-diagnosis remains a major challenge for the evaluation of any new diagnostic test for TB in children however the evidence of high mortality in this vulnerable group serves to underline the continued importance of research in this field.

## Supporting Information

Box S1 **Inclusion criteria.**
(DOC)

Box S2 **Diagnostic classifications.** Classifications in children in line with previous paediatric studies (Liebeschuetz 2004, Marais 2006, Bamford 2010) and in accordance with a recent expert consensus on TB classifications for the use in childhood TB research (Graham 2012).
(DOC)
