# Supplementary material for: QuantiFERON®-TB Gold In-Tube Performance for Diagnosing Active Tuberculosis in Children and Adults in a High Burden Setting
Source: PLoS One. 2012 Jul 12;7(7):e37851. doi: 10.1371/journal.pone.0037851 (PMC3395691; doi:10.1371/journal.pone.0037851)
Supplement: Box S1 — Inclusion criteria. (DOC) [file pone.0037851.s001.doc]

**Box S1. Inclusion criteria.**

**Children**

Children under 15 years and reported ill for ≥ 2 weeks with one or more of following:

1. Fever ≥ 2 weeks
2. Cough ≥ 2 weeks
3. Reported weight loss or failure to gain weight ≥ 2 weeks
4. Exposure to definite or probable case of TB in last 2 years
5. Admitted to hospital or contact to health facility ≥ 2 times in last 3 months.
6. Z-Score ≤-2

**Adults**

Adults, 15 years and older diagnosed with TB on the basis of positive sputum microscopy for acid fast bacteria with Ziehl-Neelsen stain and either culture or fluorescence microscopy positive.
