# Supplementary material for: QuantiFERON®-TB Gold In-Tube Performance for Diagnosing Active Tuberculosis in Children and Adults in a High Burden Setting
Source: PLoS One. 2012 Jul 12;7(7):e37851. doi: 10.1371/journal.pone.0037851 (PMC3395691; doi:10.1371/journal.pone.0037851)
Supplement: Box S2 — Diagnostic classifications. Classifications in children in line with previous paediatric studies (Liebeschuetz 2004, Marais 2006, Bamford 2010) and in accordance with a recent expert consensus on TB classifications for the use in childhood TB research (Graham 2012). (DOC) [file pone.0037851.s002.doc]

**Box S2. Diagnostic classifications.**

**Children**

**Microbiologically confirmed TB**

Clinical specimens positive for *M. tuberculosis* on solid culture or acid fast bacteria on auramine fluorescence microscopy

**Highly probably TB**

Chest x-ray highly suggestive of active tuberculosis and good clinical response

OR

Good clinical response and one of following:

- - Cervical lymphadenopathy with sinus formation
  - Abdominal mass or ascites
  - Spinal gibbus
  - Clinical picture of meningitis associated with CSF changes consistent with TB meningitis

**Possible TB**

Children who did not have confirmed or highly probable TB, but in whom active tuberculosis could not be excluded. Includes both children who were and weren’t started on anti-TB treatment.

**Not TB**

One of following:

- Spontaneous symptom resolution defined as well-being without TB treatment
- Alternative diagnosis confirmed

**Confirmed TB**

Combined classification of microbiologically confirmed TB and highly probable TB.

**Adults**

**Confirmed TB**

Microbiologically confirmed TB by either positive culture or fluorescence microscopy
